# Identification of protein functions using a machine-learning approach based on sequence-derived properties

**DOI:** 10.1186/1477-5956-7-27

**Published:** 2009-08-09

**Authors:** Bum Ju Lee, Moon Sun Shin, Young Joon Oh, Hae Seok Oh, Keun Ho Ryu

**Affiliations:** 1 Industrial Research Center, Jungwon University, 5 Dongbu-ri, Goesan-eup, Goesan-gun, Chungbuk 367-805, Republic of Korea; 2 Dept. of Computer Science, Konkuk University, 322 Danwol-Dong, Chungju-Si, Chungbuk 380-701, Republic of Korea; 3 Dept. of Computer Science, Kyungwon University, 161 Bokjung-Dong, Soojung-Gu, Seongnam-Si, Gyeonggi-Do 461-701, Republic of Korea; 4 School of Electrical and Computer Engineering, Chungbuk National University, 12 Gaeshindong, Cheongju, Chungbuk 361-763, Republic of Korea

## Abstract

**Background:**

Predicting the function of an unknown protein is an essential goal in bioinformatics. Sequence similarity-based approaches are widely used for function prediction; however, they are often inadequate in the absence of similar sequences or when the sequence similarity among known protein sequences is statistically weak. This study aimed to develop an accurate prediction method for identifying protein function, irrespective of sequence and structural similarities.

**Results:**

A highly accurate prediction method capable of identifying protein function, based solely on protein sequence properties, is described. This method analyses and identifies specific features of the protein sequence that are highly correlated with certain protein functions and determines the combination of protein sequence features that best characterises protein function. Thirty-three features that represent subtle differences in local regions and full regions of the protein sequences were introduced. On the basis of 484 features extracted solely from the protein sequence, models were built to predict the functions of 11 different proteins from a broad range of cellular components, molecular functions, and biological processes. The accuracy of protein function prediction using random forests with feature selection ranged from 94.23% to 100%. The local sequence information was found to have a broad range of applicability in predicting protein function.

**Conclusion:**

We present an accurate prediction method using a machine-learning approach based solely on protein sequence properties. The primary contribution of this paper is to propose new *PNPRD *features representing global and/or local differences in sequences, based on positively and/or negatively charged residues, to assist in predicting protein function. In addition, we identified a compact and useful feature subset for predicting the function of various proteins. Our results indicate that sequence-based classifiers can provide good results among a broad range of proteins, that the proposed features are useful in predicting several functions, and that the combination of our and traditional features may support the creation of a discriminative feature set for specific protein functions.

## Background

The need to analyse the massive accumulation of biological data generated by high-throughput human genome projects has stimulated the development of new and rapid computational methods. Computational approaches for predicting and classifying protein functions are essential in determining the functions of unknown proteins in a faster and more cost-effective manner, because experimentally determining protein function is both costly and time-consuming. Approaches based on sequence and structure comparisons play an important role in predicting and classifying the function of unknown proteins. Generally, if an unknown gene or protein sequence is identified, researchers may carry out a sequence similarity search using BLAST [1], PSI-BLAST [2], or FASTA [3] to find similar proteins or annotation information in public databases. However, proteins that have diverged from a common ancestral gene may have the same function but different sequences [4,5]. As a result, sequence similarity-based approaches are often inadequate in the absence of similar sequences or when the sequence similarity among known protein sequences is statistically weak (called the "twilight zone" or "midnight zone") [6-12]. Thus, researchers should be cautious when using this approach, because its efficiency is limited by the availability of annotated sequences in public databases and a high-similarity BLAST search result does not always imply homology [13,14]. Structure similarity-based approaches – such as Deli[15] or MATRAS [16], both of which use structure comparisons – have routinely been used to identify proteins with similar structures, because protein structural information is better conserved than sequence information [17,18]. Nevertheless, proteins with the same function can have different structures, and a structural comparison through structure determination is more difficult than a sequence comparison [9,19,20].

Recently, several researchers have developed methods for classifying and predicting protein function independent of sequence or structural alignment [5-9,20-50]. Rather than making predictions based on direct sequence or structural comparisons, these approaches use various features to predict protein function, such as protein length, molecular weight, number of atoms, grand average of hydropathicity (GRAVY), amino acid composition, periodicity, physicochemical properties, predicted secondary structures, subcellular location, sequence motifs or highly conserved regions, and annotations in protein databases. These features include statistics extracted from the protein sequence, structure, and annotation. In addition, to obtain good predictive power, various machine-learning algorithms such as support vector machines (SVMs), neural networks, naïve Bayes classifiers, and ensemble classifiers have been used to build classification and prediction models. Of these, the most widely used machine-learning algorithm for classification and prediction of protein function is SVM [5-7,22,26,27,29,31,36,39-43,45].

A method for classifying the functions of homodimeric, drug absorption, drug delivery, drug excretion, and RNA-binding proteins, among others, has been proposed by Cai *et al*. [29]. Classification of protein function was performed using an SVM statistical learning algorithm, based on features such as amino acid composition, hydrophobicity, solvent accessibility, secondary structure, surface tension, charge, polarisability, polarity, and normalised van der Waals volume. Cai *et al*. [29] found that the testing accuracy of protein classification was in the range 84–96% and suggested that amino acid composition, hydrophobicity, polarity, and charge play more critical roles than other features. Recently, Tung *et al*. [40] proposed a prediction method for ubiquitylation sites using three datasets (amino acid identity, physicochemical properties, and evolutionary information) and three machine-learning algorithms (k-nearest neighbour, SVM, and naïve Bayes). The greatest accuracy (72.19%) was obtained using SVM with 531 physicochemical properties as features. Moreover, accuracy improved from 72.19% to 84.44% when 31 physicochemical properties were selected and used based on feature selection by an informative physicochemical property mining algorithm (IPMA). In addition, Li *et al*. [45] demonstrated the ability of the SVM prediction method to identify potential drug targets. On the basis of amino acid composition, hydrophobicity, polarity, polarisability, charge, solvent accessibility, and normalised van der Waals volume, they obtained an accuracy of 84% in predicting known drug targets versus putative nondrug targets. In that study, the performance of the SVM model did not change significantly with a greater number of negative targets, as determined from experiments using various ratios of negative to positive samples.

Protein function has been predicted using naïve Bayes classifiers in several studies [21,49,50]. The FEATURE framework for recognition of functional sites in macromolecular structures was developed by Halperin *et al*. [49]. They used naïve Bayes classification to find and weigh the most informative properties that distinguish sites from nonsites, using a large number of physicochemical properties. In another study, Gustafson *et al*. [50] suggested a classification method for identification of genes essential to survival by using naïve Bayes classification. They focused on easily obtainable features such as open reading frame (ORF) size, upstream size, phyletic retention, amino acid composition, codon bias, and hydrophobicity, and they concluded that the best performing feature was phyletic retention or the presence of an orthologue in other organisms.

Neural networks are also frequently used for function prediction [23-25,36]. For example, six enzyme classes and enzymes/nonenzymes were predicted by Jensen *et al*. [24], based solely on a few meaningful features such as O-β-GlcNAcsites, *N*-linked glycosylation, secondary structure, and physicochemical properties. Function prediction was carried out using a neural network, and certain meaningful features – such as differences in secondary structures between enzymes and nonenzymes – were analysed. The discriminative ability of each feature was represented by its correlation coefficient.

Ensemble classifiers have recently become popular for protein function classification [25,35,46,47,51-55]. Zhao *et al*. [35] suggested that no single-classifier method can always outperform other methods and that ensemble classifier methods outperform other classifier methods because they use various types of complementary information. In comparing the performance of classifiers for predicting glycosylation sites, Caragea *et al*. [52] demonstrated that ensembles of SVM classifiers outperformed single SMV classifiers. In addition, Guan *et al*. [51] illustrated the benefits of using an SVM-based ensemble framework by analysing the performance of ensembles of three classifiers as a single SVM, a hierarchically corrected combination of SVMs, and naïve Bayes classifiers. Ge *et al*. [53] provided evidence that ensemble classifiers outperform single decision tree classifiers by comparing C4.5 with several ensemble classifiers (i.e. random forest, stacked generalisation, bagging, AdaBoost, LogitBoost, and MultiBoost) for classification of premalignant pancreatic cancer mass-spectrometry data. A prediction method using a domain-based random forest of decision trees to infer protein-protein interactions (PPIs) was proposed by Chen *et al*.[46]; in experiments using a *Saccharomyces cerevisiae *dataset, they showed that the random-forest method achieved higher sensitivity (79.78%) and specificity (64.38%) than maximum likelihood estimation (MLE).

These previous studies exploit direct relationships between basic protein properties and their functions to predict protein function without consideration of sequence or structural similarities. Among these studies, a wide variety of features or only a few meaningful features were selected to increase the performance of function prediction based on experience or a few heuristics. However, in most of the previous studies, features that represent subtle distinctions in small portions of protein sequences have not been sufficiently represented. Although proteins share similar structural organisations, biological properties, and sequences, small changes in amino acids of a protein sequence can result in different functions [19,20,56]. Although local information such as the presence of motifs or highly conserved regions is useful for function prediction, motif detection problems present another arduous task. Therefore, in this study, a method was developed to detect small changes in amino acids within a sequence.

The method described here is characterised by three primary features designed to address specific problems inherent in protein function prediction. First, this approach was designed to accurately predict various protein functions over a broad range of cellular components, molecular functions, and biological processes without using sequence or structural similarity information. Second, this study was designed to determine whether the use of feature selection improves prediction performance for various protein functions. In other words, does the use of more features related to the protein sequence increase the accuracy of prediction? Third, this study was designed to determine whether local information for the protein sequence is meaningful in predicting protein function, and if so, to determine which features are correlated with protein function.

In summary, a highly accurate prediction method capable of identifying protein function is proposed. One of the advantages of this method is that it requires only the protein sequence for feature extraction; information *vis-à-vis *predicted features or structural properties is not required. In addition, four features that represent subtle differences in local regions of the protein sequence – differences due to positively and negatively charged residues – are introduced. A total of 484 features, including 451 traditional features and 33 features introduced in this study, were used to predict 11 protein functions. We applied two machine-learning algorithms (i.e. SVM and random forests) with and without feature selection to the data set to predict protein function. The prediction performance for each protein function was evaluated, and the features most relevant to prediction of specific protein functions were determined.

## Methods

### Data preparation

To predict the functions of a variety of proteins from a broad range of cellular components, molecular functions, and biological processes, 16,618 positive sample sequences and 35,619 negative sample sequences were collected and comprised the dataset. The dataset included positive and negative samples for 11 protein functions selected from the Swiss-Prot database [57] using the SRS program [58]. Positive samples are sample sequences associated with a specific protein function and were labelled as belonging to that class of proteins. Negative samples for each protein class were selected from proteins that do not belong to that class and from enzyme families such as oxidoreductases, hydrolases, lyases, and isomerases. For example, the composition of the negative sample set for fatty acid metabolism is shown in Table 1. Proteins that consisted of fewer than 30 amino acids were excluded from the dataset. In the feature extraction step, proteins that had missing values were also excluded. For hypothetically or automatically annotated sequences in protein databases such as GenBank and Swiss-Prot, the percentage of incorrect annotations is not known, because the annotations do not include a description of the specific methodology used for sequence analysis; this sometimes yields incorrect search results [14]. However, Swiss-Prot incorporates corrections provided by user forums and communities [13]; therefore, protein sequences from the Swiss-Prot database were used in the present study.

**Table 1:** Negative samples for the fatty acid metabolism protein class

Protein class	Number of proteins
Transport	637
Transcription	538
Gluconate utilisation	60
Amino acid biosynthesis	393
DNA-binding	486
Acetylcholine receptor inhibitor	103
G-protein coupled receptor	220
Guanine nucleotide-releasing factor	370
Fibre protein	47
Transmembrane	351
Oxidoreductases	58
Hydrolases	75
Isomerases	50
Lyases	72
Other proteins	354

### Feature extraction from protein sequences

A total of 484 features were extracted solely from the protein sequences described in this study. These features included traditional features adopted from previous studies [7,30-32] and new features extracted using the novel method developed in this study. Among the traditional features, 34 were extracted from the Swiss-Prot protein sequences [57] using the ProtParam tool [59]. Traditional features consisted of amino acid composition, protein length, number of atoms, molecular weight, GRAVY, and theoretical isoelectric point (pI), among others. In addition, two ways of using positively charged residues (i.e. lysine and arginine or histidine, lysine, and arginine), the percent composition of each amino acid pair, and 17 features based on physicochemical properties (i.e. 16 properties adopted from Syed *et al*. [20] and one additional property) were calculated.

The importance of negatively/positively charged residues in protein function has been described in several studies [20,23,24,38,60]. The 20 standard amino acids are divided into negatively charged residues, positively charged residues, and neutral residues according to their pI. Negatively charged residues (aspartic acid and glutamic acid) have lower pIs, while positively charged residues (arginine and lysine) have higher pIs. Oppositely charged residues attract, while similarly charged residues repel each other. To account for subtle differences that occur in small regions of the protein sequences, features representing the percentage change in charged residues as well as the distribution of charged residues were designed and computed.

The method used to identify these new features is simple. *PPR *was calculated using the following equation:

(1)

where *#AA *is the total number of amino acids in a sequence and *#PP *is the total number of continuous changes from one positively charged residue to the next positively charged residue in each protein sequence. Similar to *PPR*, *NNR *was calculated using the following equation:

(2)

where *#NN *is the total number of continuous changes from one negatively charged residue to the next negatively charged residue. *PNPR *was calculated using the following equation:

(3)

where *#PNP *is the total number of continuous changes from a positively charged residue to the next negatively charged residue or vice versa. Finally, *Dist*_(*x*, *y*) _is the distribution function for *PP*, *NN*, or *PNP *in the interval from *x *to *y *in the sequence, with the stipulation that *x *<*y*. *PPRDist*_(*x*, *y*) _was defined as follows:

(4)

where *#PP*_(*x*, *y*) _is the total number of *PP *occurrences in the interval from *x *to *y*. Similarly, *NNRDist*_(*x*, *y*) _was computed as follows:

(5)

where *#NN*_(*x*, *y*) _is the total number of *NN *occurrences in the interval from *x *to *y*.*PNPRDist*_(*x*, *y*) _was computed as follows:

(6)

where *#PNP*_(*x*, *y*) _is the total number of *PNP *occurrences of the interval from *x *to *y*. These features provide local information on a protein sequence based on the values of *x *and *y*. For example, the alcohol dehydrogenase1A protein (Swiss-Prot:P07327) consists of 375 amino acids. Let us assume that the *x *value is the 76th amino acid (21%), the *y *value is the 113th amino acid (30%), and the value of *PNP *is 4. *PNPRDist*_(21,30) _is thus (4/375) × 100 = 1.06667. We believe these features are important because slight regional differences among similar proteins exist in sequences within the same family. Certain protein functions are determined by a few residues within a small part of the sequence [61]. A total of 33 features were generated based on the above formulae, dividing the sequence length into 10 local regions, specifically, *PPR *(1), *NNR *(1), *PNPR *(1), *PPRDist*_(*x*, *y*) _(10), *NNRDist*_(*x*, *y*) _(10), and *PNPRDist*_(*x*, *y*) _(10). All the traditional and novel features used in this study are described in detail in Table 2.

**Table 2:** Features used for protein function classification

	Feature	Description	Dimension
1	Number of amino acids	Number of residues in each protein	1
2	Molecular weight	Molecular weight of the protein	1
3	Theoretical pI	The pH at which the net charge of the protein is zero (isoelectric point)	1
4	Amino acid composition	Percentage of each amino acid in the protein	20
5	Positively charged residue_2	Percentage of positively charged residues in the protein (lysine and arginine)	1
6	Positively charged residue_3	Percentage of positively charged residues in the protein (histidine, lysine, and arginine)	1
7	Number of atoms	Total number of atoms	1
8	Carbon	Total number of carbon atoms in the protein sequence	1
9	Hydrogen	Total number of hydrogen atoms in the protein sequence	1
10	Nitrogen	Total number of nitrogen atoms in the protein sequence	1
11	Oxygen	Total number of oxygen atoms in the protein sequence	1
12	Sulphur	Total number of sulphur atoms in the protein sequence	1
13	Extinction coefficient_All	Amount of light a protein absorbs at a certain wavelength (assuming ALL Cys residues appear as half cysteines)	1
14	Extinction coefficient_No	Amount of light a protein absorbs at a certain wavelength (assuming NO Cys residues appear as half cysteines)	1
15	Instability index	The stability of the protein	1
16	Aliphatic index	The relative volume of the protein occupied by aliphatic side chains	1
17	GRAVY	Grand average of hydropathicity	1
18	*PPR*	Percentage of continuous changes from positively charged residues to positively charged residues	1
19	*NNR*	Percentage of continuous changes from negatively charged residues to negatively charged residues	1
20	*PNPR*	Percentage of continuous changes from positively charged residues to negatively charged residues or from negatively charged residues to positively charged residues	1
21	*NNRDist*_(*x*, *y*)_	Percentage of *NNR *from *x *to *y *(local information)	10
22	*PPRDist*_(*x*, *y*)_	Percentage of *PPR *from *x *to *y *(local information)	10
23	*PNPRDist*_(*x*, *y*)_	Percentage of *PNPR *from *x *to *y *(local information)	10
24	Charged	Physicochemical property	1
25	Negatively charged residues	Percentage of negatively charged residues in the protein	1
26	Polar	Physicochemical property	1
27	Aliphatic	Physicochemical property	1
28	Aromatic	Physicochemical property	1
29	Small	Physicochemical property	1
30	Tiny	Physicochemical property	1
31	Bulky	Physicochemical property	1
32	Hydrophobic	Physicochemical property	1
33	Hydrophobic and aromatic	Physicochemical properties	1
34	Neutral, weakly and hydrophobic	Physicochemical properties	1
35	Hydrophilic and acidic	Physicochemical properties	1
36	Hydrophilic and basic	Physicochemical properties	1
37	Acidic	Physicochemical property	1
38	Polar and uncharged	Physicochemical properties	1
39	Amino acid pair ratio	Percentage compositions for each of the 400 possible amino acid dipeptides	400
	Total		484

### Feature selection

Feature selection is an important step in developing an accurate classification method. There are many redundant and/or irrelevant features in real-world problems, and various approaches have been developed to address these features. The primary goals of feature selection [62-65] are to gain a more thorough understanding of the underlying processes influencing the data and to identify discriminative and useful features for classification and prediction. In addition, classification and prediction performance can be improved by avoiding overfitting. Although additional features provide more information and could potentially improve classification performance, a greater number of features also adds difficulty in building a classifier. For *n *features there are *n*^2 ^possible feature subsets; therefore, to achieve optimal performance, it is necessary to generate all possible subsets and examine their performance.

Various feature selection methods have been developed to select an optimal feature set and analyse the discriminatory power of each feature. Feature subset selection techniques can be organised into two categories: filter and wrapper methods. Filter methods, which apply statistical approaches without any information on the classification algorithm, are used to select a specific subset of potentially discriminating features. Wrapper methods use a machine-learning algorithm, called a perfect "black box," to assess the quality of a feature subset. For this study, correlation-based feature selection (CFS) [34,65] was used to select a subset of discriminative features. CFS was chosen for the following reasons. First, when the number of features is large, filter methods are faster than wrapper methods because the former do not require the use of learning machines. In addition, filter methods can be used as a preprocessing step to reduce space dimensionality and preclude overfitting. Second, selection and evaluation of a subset of features is preferable to individually important features because a superior classifier can be constructed from features that interact or by a combination of many features that together have discriminatory power. Even if one or two features are not useful alone, these features may be valuable in combination with other features and thus improve the discriminatory performance of a classifier [64].

CFS is a filter method. It uses a search algorithm, along with a function for evaluating the merit of a feature subset, based on the hypothesis that "a good feature subset contains features highly correlated with the class, yet uncorrelated with each other" [65]. This method evaluates subsets of features, rather than individual features, as discussed above. At the core of the CFS is the subset evaluation heuristic. It eliminates irrelevant features, as they will be poor predictors of classes. In addition, redundant features are identified that will be highly correlated with one or more other features. A heuristic search to traverse the space of the feature set is conducted, and the subset with the highest merit found during the search process is reported. The subset with the highest *merit *preserves the most important features – those that are highly correlated with the class and have low inter-correlation with one another. This subset is then used to reduce dimensionality. CFS is described in greater detail elsewhere [65].

In the present study, many features were discarded during the feature subset selection procedure using CFS. *Merit *was calculated using the following equation:

(7)

Where *Merit*_*s *_is the score of a feature subset *S *that comprises *k *features,  is the average correlation between the individual features and the class, and  is the average inter-correlation among the features. The features selected by CFS for each class are listed in Table 3.

**Table 3:** Features selected by CFS for each protein class

Protein class	Selected features
Transport	R, G, H, I, M, positively charged residue_3, carbon, CC, CD, CE, CH, CK, CN, CQ, CW, CY, FM, GW, HC, HR, IC, IG, LF, LG, LM, MF, MM, MQ, PC, QC, SC, TC, WD, YH, polar, hydrophobic, hydrophobic and aromatic, hydrophilic and basic

Transcription	D, C, Q, F, V, positively charged residue_3, sulphur, extinction coefficient_all, instability index, aliphatic index, GRAVY, *NNR*, *PNPR*, *PPRDist*_(41,50)_, CC, CF, CV, CW, CY, DD, DE, EE, EF, EH, EL, FC, FF, FW, GC, HD, HH, IF, LT, MN, QQ, TL, TW, VV, WI, WV, WW, WY, charged, polar, aliphatic, aromatic, hydrophobic and aromatic, hydrophilic and acidic, hydrophilic and basic, acidic, polar and uncharged

Translation	NumOfAAs, D, L, hydrogen, GRAVY, *PPR*, *NNR*, *NNRD*_(11,20)_, *PPRD*_(31,40)_, *PNPRD*_(41,50)_, *PPRD*_(51,60)_, *PNPRD*_(81,90)_, *NNRD*_(91,100)_, *PNPRD*_(91,100)_, AA, AG, AH, AM, AQ, CC, CE, CN, CP, DE, DH, EE, EG, EQ, FD, FK, FQ, FW, GC, GV, GW, GY, HI, IC, IP, IY, KE, KK, KR, KS, KW, LG, LK, LT, LV, LW, MM, MW, NH, PE, PK, PT, PY, QF, QN, RN, SD, TG, TK, VA, VG, VL, WC, WE, WG, WK, YD, YS, YV, charged, aliphatic, hydrophilic and acidic

Gluconate utilisation	Positively charged residue_3, instability index, aliphatic index, *PNPRDist*_(11,20)_, *PPRDist*_(21,30)_, *PPRDist*_(31,40)_, *PPRDist*_(81,90)_, *PPRDist*_(91,100)_, AG, AH, AV, AW, AY, CC, CI, DG, DI, DR, EG, EW, FH, FL, FP, GC, GE, GF, GI, GK, GM, GP, GR, HN, IG, KL, KM, KW, LI, LM, MG, MM, MQ, MV, PC, PK, PN, PP, SR, SY, TD, VF, VM, WN, WR, WT, YS, YV, aromatic, hydrophilic and acidic, polar and uncharged

Amino acid biosynthesis	NumOfAAs, theoretical pI, D, C, G, S, sulphur, instability index, aliphatic index, GRAVY, *PPR*, *NNR*, *NNRD*_(11,20)_, *PNPRD*_(21,30)_, *NNRD*_(91,100)_, CN, DC, DM, EC, EW, FP, FW, FY, GA, HP, IN, LC, MW, NF, NW, PC, PP, PS, QM, RC, RD, SC, WF, WG, WM, WN, WW, YR, YY, charged, aliphatic, tiny, bulky, hydrophobic, hydrophobic and aromatic, hydrophilic and acidic, acidic

Fatty acid metabolism	NumOfAAs, R, D, C, Q, E, G, I, F, S, negatively charged residue, positively charged residue_3, instability index, aliphatic index, GRAVY, *NNR*, *PNPR*, *PPRD*_(00,10)_, *PNPRD*_(71,80)_, *PPRD*_(81,90)_, *PNPRD*_(81,90)_, *PPRD*_(91,100)_, *NNRD*_(91,100)_, AH, AR, CG, CI, DC, DN, DR, EC, EY, FA, FP, GA, GG, GL, GW, HH, HI, HM, HN, HP, HT, IR, IW, KA, KH, LF, LL, MC, MG, MH, MM, MR, NA, NP, PA, PC, PP, PR, PY, QM, QN, QP, RK, RR, RS, SM, SY, TD, TR, TS, TW, VQ, VW, WG, WP, WQ, WS, WW, YG, YI, YW, YY, charged, aliphatic, hydrophobic, hydrophilic and acidic, acidic

Acetylcholine receptor inhibitor	Molecular weight, C, M, *PNPRDist*_(00,10)_, *NNRDist*_(11,20)_, *NNRDist*_(71,80)_, AN, AT, CA, CC, CF, CN, CP, CS, DA, DF, DP, DS, EA, EI, ES, ET, FL, GC, HI, HQ, IC, II, IR, IT, KC, KE, KF, KL, KT, LD, LE, LN, LP, LQ, MK, NC, NV, RI, TC, VK, VN, VS, WC, YD, YT, tiny

G-protein coupled receptor	Theoretical pI, D, C, Q, E, G, K, F, S, T, negatively charged residue, positively charged residue_3, sulphur, *PNPR*, *PNPRDist*_(11,20)_, *NNRDist*_(71,80)_, CC, CF, CH, CW, CY, FC, FI, FL, GQ, IC, IW, IY, LC, MW, SC, WG, WV, WY, aromatic, tiny, bulky, hydrophobic and aromatic, acidic

Guanine nucleotide-releasing factor	A, Q, H, I, V, positively charged residue_2, positively charged residue_3, oxygen, instability index, aliphatic index, GRAVY, *PPR*, *NNR*, *NNRDist*_(00,10)_, *PPRDist*_(11,20)_, *PNPRDist*_(21,30)_, *NNRDist*_(31,40)_, *PNPRDist*_(51,60)_, *PNPRDist*_(61,70)_, *NNRDist*_(91,100)_, CQ, DC, DH, EC, ED, EE, EP, EW, FN, HC, HD, HE, HH, HK, HM, HW, IV, KW, LE, LG, LK, MF, MI, PN, QC, QD, QE, QW, RL, SE, SP, TW, VG, VI, VV, WC, WD, WE, WF, WK, WS, WY, YW, hydrophobic, hydrophilic and acidic, hydrophilic and basic, acidic, polar and uncharged, polar

Fibre protein	G, M, T, positively charged residue_2, *NNRDist*_(81,90)_, DN, ER, FN, GD, GG, GN, GQ, GT, IN, IP, LC, LL, LT, NA, NG, NT, PF, SQ, TA, TG, TN, TW, WK, WN, charged, polar and uncharged

Transmembrane	Theoretical pI, D, C, L, S, W, negatively charged residue, extinction coefficient_all, instability index, GRAVY, *NNR*, *PNPR*, *PPRDist*_(71,80)_, AD, CC, CW, DA, EA, FC, FL, FW, LK, LL, LW, MW, PC, PP, SC, SL, TW, VD, WW, tiny, bulky, acidic

Finding and identifying important features that discriminate protein function is an arduous task; however, it is possible to evaluate which discriminative features are important using feature subset selection methods. For instance, using the CFS method, for the transmembrane protein class (Table 4, last row), the number of traditional features selected was 33 of 451 features and the number of new features selected was 3 of 33 features. Selection rates were then calculated as (33/451) × 100 = 7.76% and (3/33) × 100 = 9.09%. Several of the new features were preserved in every subset selected by the CFS method, except for the transport class. Therefore, it can be inferred that these features are highly correlated with the class and that they have low inter-correlation with each other.

**Table 4:** Selection ratios for traditional and new features in the CFS method

Protein class	Number of selected features	*Merit *value	Traditional features (n = 451)	New features (n = 33)
Transport	38	0.302	8.43%	0%
Transcription	51	0.387	11.31%	9.09%
Translation	76	0.499	16.85%	27.27%
Gluconate utilisation	59	0.59	13.08%	15.15%
Amino acid biosynthesis	52	0.309	11.53%	15.15%
Fatty acid metabolism	90	0.303	19.96%	24.24%
Acetylcholine receptor inhibitor	52	0.974	11.53%	9.09%
G-protein coupled receptor	39	0.487	8.65%	9.09%
Guanine nucleotide-releasing factor	69	0.36	15.30%	27.27%
Fibre protein	31	0.481	6.87%	3.03%
Transmembrane	35	0.443	7.76%	9.09%

The *merit *value is the highest merit calculated for an optimal subset of the features for each class. The selected features are highly correlated with the class and have low inter-correlation with each other.

### Support vector machines and random forests

In the preprocessing step, numeric features were discretised via an MDL-based discretisation method [66]. Each dataset was randomly split into a training set (90%) and a blind test set (10%). The numbers of negative and positive samples in the training and test data sets are shown in Table 5. Validation was performed by 10-fold cross-validation on the training set, and test results for the blind test process were obtained using a separate test dataset. No sample was included in both the training and testing sets. We present only the average performance of the 10-fold cross-validation process because the Weka tool [67] does not provide experimental results for each iteration of *k*-fold cross-validation.

**Table 5:** Accuracy of predictions using training and blind test datasets with the SVM and random forest methods

Category	Protein class	Training set		Test set		SVM_FF		SVM_CFS		RF_FF		RF_CFS	
		
		Positive	Negative	Positive	Negative	Train	Test	Train	Test	Train	Test	Train	Test
Biological process	Transport	2,824	3,583	298	414	73.26	71.34	94.38	93.53	93.14	92.41	94.66	**94.24**
	Transcription	3,644	3,872	415	421	87.78	85.04	96.62	**96.65**	94.25	94.61	94.65	94.73
	Translation	139	1,886	16	210	98.81	**98.67**	98.37	97.78	97.87	96.90	98.07	97.34
	Gluconate utilisation	53	420	7	46	98.73	98.11	98.94	98.11	97.04	98.11	98.30	**100**
	Amino acid biosynthesis	2,769	3,970	289	460	73.55	76.63	90.28	92.12	95.69	**96.12**	96.29	**96.12**
	Fatty acid metabolism	601	3,445	81	369	90.58	87.55	94.19	92	95.99	94.88	96.93	**95.77**

Molecular function	Acetylcholine receptor inhibitor	93	1,840	10	205	100	99.53	100	**100**	100	**100**	100	**100**
	G-protein coupled receptor	2,571	3,828	263	448	76.04	77.07	98.76	**98.17**	96.62	97.74	97.60	97.46
	Guanine nucleotide-releasing factor	335	3,994	35	446	98.96	98.75	99.51	**98.96**	98.49	**98.96**	98.98	98.54

Cellular component	Fibre protein	42	1,266	6	140	99.84	**99.31**	99.92	**99.31**	99.38	**99.31**	99.84	98.63

Domain	Transmembrane	1,904	3,930	223	426	80.01	79.81	97.15	**97.84**	96.02	**97.84**	96.46	97.38

The accuracy of predictions using the training dataset was determined when building the classification model using 10-fold cross validation, and the accuracy of predictions using the test dataset was determined using the built model. The accuracies of predictions for all the training and test datasets are presented to demonstrate a good balance between overfitting and underfitting. Positive: number of positive samples; negative: number of negative samples; FF: full features; CFS: correlation-based feature subset selection method. The bold values mean the highest values among four methods.

The abilities of the SVM and random forest techniques to predict and classify protein functions have recently been enhanced and found to be superior to other classification algorithms [5,6,22,26,29,33,46,47]. SVM is essentially a two-class classifier, although the classifier can be extended to multiclass classifications. In this model, each object is mapped to a point in a high-dimensional space, where each dimension corresponds to a feature. The coordinates of the point are the frequencies of the features in their corresponding dimensions. In the training step, SVM learns the maximum-margin hyper-planes separating each class. In the testing step, a new object is classified by mapping it onto a point in the same high-dimensional space, divided by the hyper-plane that was learned in the training step.

Recently, the random forest method [68] has also become popular for protein function prediction. Random forests is a classification algorithm that employs an ensemble of classification trees that each use several bootstrap samples of training data and a randomly selected subset of features. The basic random forest method, using unpruned decision trees, selects features at random at each decision node. The final classification is obtained by combining the results of the trees via voting.

To identify protein functions in this study, LibSVM [69,70] and random forests [68] (available at Weka [67]) were used as the classification algorithms. The type of SVM used was a C-SVC machine, and the kernel was a radial basis function (RBF). The cost parameter was set at 4 and the other parameters were fixed at the default values. The cost parameter used in the training process was selected from {0.5, 1, 2, 4, 6, 8, 10, 12}. For the datasets used in this study, the RBF was found to provide the best results. In the random forest method without feature selection analysis, the number of trees was 10 and the number of features was 9. In the random forest method with feature selection analysis, the number of trees was 10 and the number of features was 6 or 7 because the number of features selected by the feature selection method was small.

### Performance evaluation criteria

The following measures were used to assess the performance of the classifiers used in this study: accuracy, sensitivity, F-measure, Matthew's correlation coefficient (MCC) [71,72], and the area under the receiver operating characteristic curve (AUC) [42,71]. A trade-off between sensitivity and specificity was observed as the prediction threshold was varied. AUC is an effective means of comparing the overall prediction performance of different methods because it provides a single measure of overall threshold-independent accuracy. An AUC and MCC of 1 indicate perfect prediction accuracy. These measures are defined as follows:

(8)

(9)

(10)

(11)

(12)

where *TP *is the number of true positives, *FP *is the number of false positives, *TN *is the number of true negatives, *FN *is the number of false negatives, and *recall *is equivalent to the sensitivity [21,73]. The formula for the AUC of a classifier is as follows:

(13)

where *S*_0 _= ∑*r*_*i*_, *r*_*i *_is the rank of the *i*th positive sample in the ranked list, *n*_0 _is the number of positive samples, and *n*_1 _is the number of negative samples [74,75].

## Results and discussion

### Performance of the four classification methods

One of the goals of our experiment was to find a more discriminative and smaller feature set for specific function prediction, based solely on sequence-based features. Therefore, we initially gathered numerous features solely from the protein sequence. The features that were redundant or irrelevant were then removed by feature selection. After feature selection, the remaining number of features was small, while the accuracy of function classification was greater than that of the full-feature set. The selected features and the selection rates for the traditional features and our new features are listed in Tables 3 and 4.

**Figure 1:**
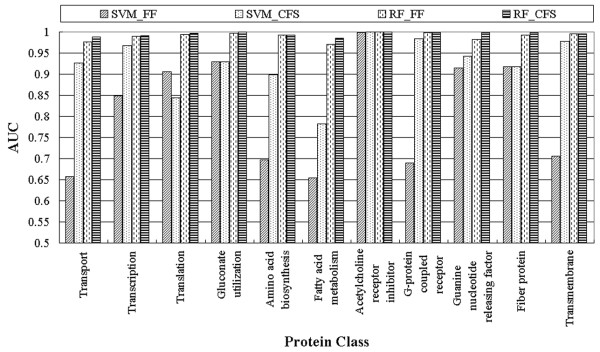
**Area under the ROC curves for the four methods for each protein class**.

A summary of the performance of the four methods in classifying the 11 protein classes is provided in Table 5 and Figure 1. Among all the methods, SVM without feature selection (SVM_FF) required more model-building time and had the lowest performance. However, this method did obtain the highest accuracy in two of the blind tests, for translation and fibre proteins (2 of the 11 protein classes).

SVM with feature selection (SVM_CFS) slightly outperformed the random forest method with and without feature selection (RF_CFS and RF_FF, respectively) and significantly outperformed SVM_FF. Given that more than one method had equal accuracy for some classes, the SVM_CFS method had the highest accuracy for classifying transcription, acetylcholine receptor inhibitor, G-protein coupled receptor, guanine nucleotide-releasing factor, fibre, and transmembrane proteins (6 of the 11 protein classes).

The random forest method without feature selection (RF_FF) had the highest accuracy for the following blind test sets: amino acid biosynthesis, acetylcholine receptor inhibitor, guanine nucleotide-releasing factor, fibre, and transmembrane proteins (5 of the 11 protein classes).

The random forest method with feature selection (RF_CFS) had the highest accuracy for classifying transport, gluconate utilisation, amino acid biosynthesis, fatty acid metabolism, and acetylcholine receptor inhibitor proteins (5 of the 11 protein classes). Although both the RF_FF and RF_CFS methods had the highest accuracy for five protein classes, the performance of the RF_CFS method was better than that of the RF_FF method in terms of cost-effectiveness because a reduced-dimensional model was produced.

After careful analysis of the selected feature subsets and their performance in these experiments, the use of feature selection was found to improve classifier performance, as indicated in Table 5 and Figure 1. When comparing the SVM_FF and SVM_CFS methods, translation was the only protein class for which the SVM_FF method (AUC of 0.906, accuracy of 98.67) performed better than the SVM_CFS method (AUC of 0.844, accuracy of 97.78). For all the other classifications, the use of feature selection improved classifier performance. For classification of transport, transcription, amino acid biosynthesis, G-protein coupled receptor, and transmembrane proteins, both accuracy levels and AUCs significantly improved when feature selection was used. For example, the accuracy of transmembrane protein classification improved (79.81 to 97.84) and the AUC increased (0.706 to 0.978). In addition, the number of features used for classification was 35 of 484. For the G-protein coupled receptor, 39 of 484 features were included, and the accuracy of the classification was dramatically improved by feature selection (77.07 to 98.17), as was the AUC (0.69 to 0.984).

Although the accuracy of the random forest method was not significantly improved by feature selection, the AUC value for RF_CFS was slightly higher than that for RF_FF, except for the amino acid biosynthesis, acetylcholine receptor inhibitor, and G-protein coupled receptor classes. The larger the AUC, the better is the performance of the model. By comparing the AUCs averaged over all 11 protein classes, the RF_CFS method was found to outperform the other methods (i.e. 0.995). Therefore, applying CFS to the dataset yielded improved performance and a more compact set of features.

For a more detailed evaluation of all the methods, several performance measures were applied. Detailed results for each method are presented in Tables 6, 7, 8, and 9, with a focus on sensitivity, specificity, F-measure, and MCC. The consistent performance of each method for predicting the protein functions of the 11 protein classes in both the 10-fold cross-validation test and the blind test demonstrates the validity of our methods: SVM_FF, ± 3.0; SVM_CFS, ± 2.1; RF_FF ± 1.8; and RF_CFS, ± 1.2 (± refers to the difference in accuracy between the training step and the blind test step). These results indicate that our models have good predictive power in discriminative testing processes.

**Table 6:** Detailed results of SVM without feature selection (SVM_FF)

Protein class	Sensitivity	Specificity	F-measure	MCC
Transport	31.54	100	0.48	0.46
Transcription	71.08	98.81	0.83	0.73
Translation	81.25	100	0.90	0.9
Gluconate utilisation	85.71	100	0.92	0.92
Amino acid biosynthesis	39.45	100	0.57	0.53
Fatty acid metabolism	30.86	100	0.47	0.52
Acetylcholine receptor inhibitor	100	99.51	0.95	0.95
G-protein coupled receptor	38.02	100	0.55	0.53
Guanine nucleotide-releasing factor	82.86	100	0.91	0.9
Fibre protein	83.33	100	0.91	0.91
Transmembrane	41.26	100	0.58	0.56

MCC: Matthew's correlation coefficient

**Table 7:** Detailed results of SVM with feature selection (SVM_CFS)

Protein class	Sensitivity	Specificity	F-measure	MCC
Transport	87.58	97.83	0.92	0.87
Transcription	98.31	95.01	0.97	0.93
Translation	68.75	100	0.82	0.82
Gluconate utilisation	85.71	100	0.92	0.92
Amino acid biosynthesis	79.58	100	0.89	0.84
Fatty acid metabolism	56.79	99.73	0.72	0.71
Acetylcholine receptor inhibitor	100	100	1.00	1.00
G-protein coupled receptor	99.24	97.54	0.98	0.96
Guanine nucleotide-releasing factor	88.57	99.78	0.93	0.92
Fibre protein	83.33	100	0.91	0.91
Transmembrane	97.76	97.89	0.97	0.95

MCC: Matthew's correlation coefficient

**Table 8:** Detailed results of the random forest method without feature selection (RF_FF)

Protein class	Sensitivity	Specificity	F-measure	MCC
Transport	87.58	95.89	0.84	0.91
Transcription	96.87	92.4	0.89	0.95
Translation	56.25	100	0.74	0.72
Gluconate utilisation	85.71	100	0.92	0.92
Amino acid biosynthesis	96.89	95.65	0.92	0.95
Fatty acid metabolism	71.6	100	0.82	0.84
Acetylcholine receptor inhibitor	100	100	1.00	1.00
G-protein coupled receptor	95.44	99.11	0.95	0.97
Guanine nucleotide-releasing factor	85.71	100	0.92	0.92
Fibre protein	83.33	100	0.91	0.91
Transmembrane	95.07	99.3	0.95	0.97

MCC: Matthew's correlation coefficient

**Table 9:** Detailed results of the random forest method with feature selection (RF_CFS)

Protein class	Sensitivity	Specificity	F-measure	MCC
Transport	90.27	97.10	0.93	0.88
Transcription	96.63	92.87	0.95	0.90
Translation	62.50	100.00	0.77	0.78
Gluconate utilisation	100.00	100.00	1.00	1.00
Amino acid biosynthesis	95.85	96.30	0.95	0.92
Fatty acid metabolism	77.78	99.73	0.87	0.85
Acetylcholine receptor inhibitor	100.00	100.00	1.00	1.00
G-protein coupled receptor	96.58	97.99	0.97	0.95
Guanine nucleotide-releasing factor	85.71	99.55	0.90	0.89
Fibre protein	66.67	100.00	0.80	0.81
Transmembrane	94.62	98.83	0.96	0.94

MCC: Matthew's correlation coefficient

Although good performance with the proposed new features was obtained using feature selection, we performed an additional experiment to demonstrate the usefulness of the proposed features in a clear and simple way, without relying on feature selection. The additional experiments were carried out using the 451 traditional features versus the 33 proposed features under the same conditions as the above experiments, and the performance of classification was compared (Tables 10 and 11). In the performance comparison with SVM, classification using only the 33 proposed features outperformed the 451 traditional features for 5 of the 11 protein classes (transport, amino acid biosynthesis, fatty acid metabolism, G-protein coupled receptor, and transmembrane). In the performance comparison with the random forest method, classification using only the 33 proposed features was superior or equal to use of the 451 traditional features for 4 of the 11 protein classes (translation, gluconate utilisation, fatty acid metabolism, and acetylcholine receptor inhibitor).

**Table 10:** Comparative performance of the novel feature set and traditional feature set using SVM

Protein class	Novel feature set (33 features)					Traditional feature set (451 features)				
	
	Training Accuracy	Test accuracy	Sensitivity	Specificity	AUC	Training accuracy	Test accuracy	Sensitivity	Specificity	AUC
Transport	75.0273	**73.31**	36.2	100	0.681	73.2636	72.19	33.6	100	0.668
Transcription	87.9723	88.15	99.3	77.2	0.882	92.5625	**97.36**	98.3	96.4	0.974
Translation	97.0864	97.34	62.5	100	0.813	98.8642	**98.67**	81.3	100	0.906
Gluconate utilisation	96.8288	96.22	71.4	100	0.857	98.7315	**98.11**	85.7	100	0.929
Amino acid biosynthesis	74.8627	**77.83**	42.6	100	0.713	73.5272	77.43	41.5	100	0.708
Fatty acid metabolism	92.4123	**90.22**	45.7	100	0.728	90.5586	87.77	32.1	100	0.66
Acetylcholine receptor inhibitor	98.448	99.06	80	100	0.9	100	**99.53**	100	99.5	0.998
G-protein coupled receptor	78.6998	**80.87**	48.3	100	0.741	76.1838	77.35	38.8	100	0.694
Guanine nucleotide-releasing factor	97.4359	97.92	77.1	99.6	0.883	98.8681	**98.75**	82.9	100	0.914
Fibre protein	96.789	95.89	0	100	0.5	99.8471	**99.31**	83.3	100	0.917
Transmembrane	85.2931	**85.67**	58.3	100	0.791	79.8937	80.58	43.5	100	0.717

AUC: Area under the curve.

**Table 11:** Comparative performance of the novel feature set and traditional feature set using the random forest

Protein class	Novel feature set (33 features)					Traditional feature set (451 features)				
	
	Training accuracy	Test accuracy	Sensitivity	Specificity	AUC	Training accuracy	Test accuracy	Sensitivity	Specificity	AUC
Transport	91.3688	90.30	86.6	93	0.968	92.9764	**93.39**	89.9	95.9	0.975
Transcription	90.9659	91.26	93.7	88.8	0.98	94.4252	**95.33**	96.4	94.3	0.99
Translation	97.679	**97.78**	68.8	100	0.95	98.0741	**97.78**	68.8	100	0.996
Gluconate utilisation	96.4059	**98.11**	85.7	100	0.997	97.2516	**98.11**	85.7	100	0.992
Amino acid biosynthesis	93.7676	94.52	91.7	96.3	0.983	94.836	**95.46**	94.8	95.9	0.991
Fatty acid metabolism	95.7242	**94.44**	72.8	99.2	0.97	96.2926	94	69.1	99.5	0.964
Acetylcholine receptor inhibitor	99.6896	**100**	100	100	1	99.8965	**100**	100	100	1
G-protein coupled receptor	94.4679	95.92	94.3	96.9	0.991	96.8745	**97.18**	94.7	98.7	0.993
Guanine nucleotide-releasing factor	96.7429	96.67	62.9	99.3	0.956	98.4985	**97.92**	74.3	99.8	0.992
Fibre protein	97.4771	95.89	33.3	98.6	0.798	99.2355	**99.31**	83.3	100	0.998
Transmembrane	93.555	93.52	87.4	96.7	0.978	95.9719	**97.53**	94.2	99.3	0.995

AUC: Area under the curve.

### Meaningful features for protein function prediction

**Figure 2:**
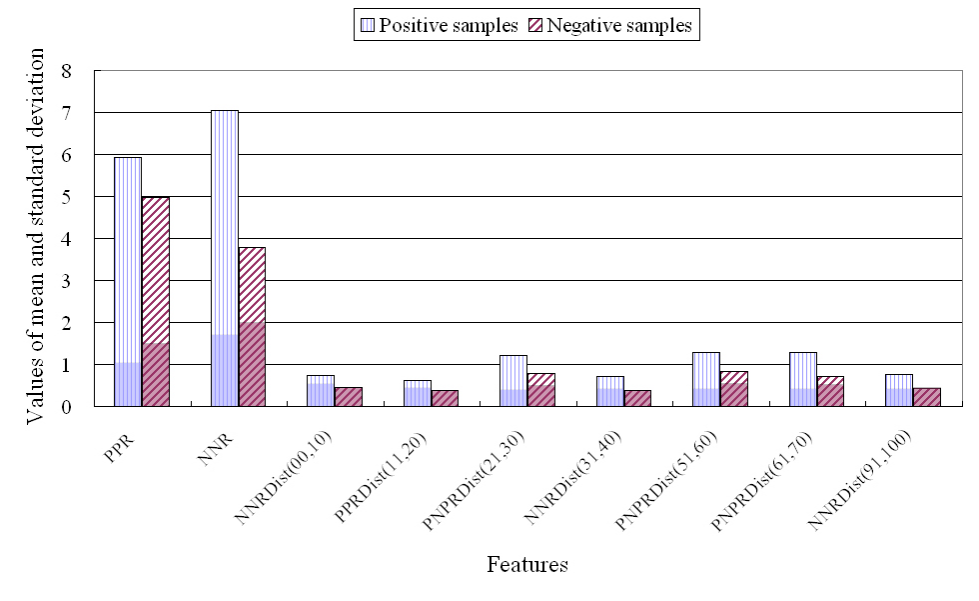
**Comparison of nine features used for classification of guanine nucleotide-releasing factor versus negative proteins**.

**Figure 3:**
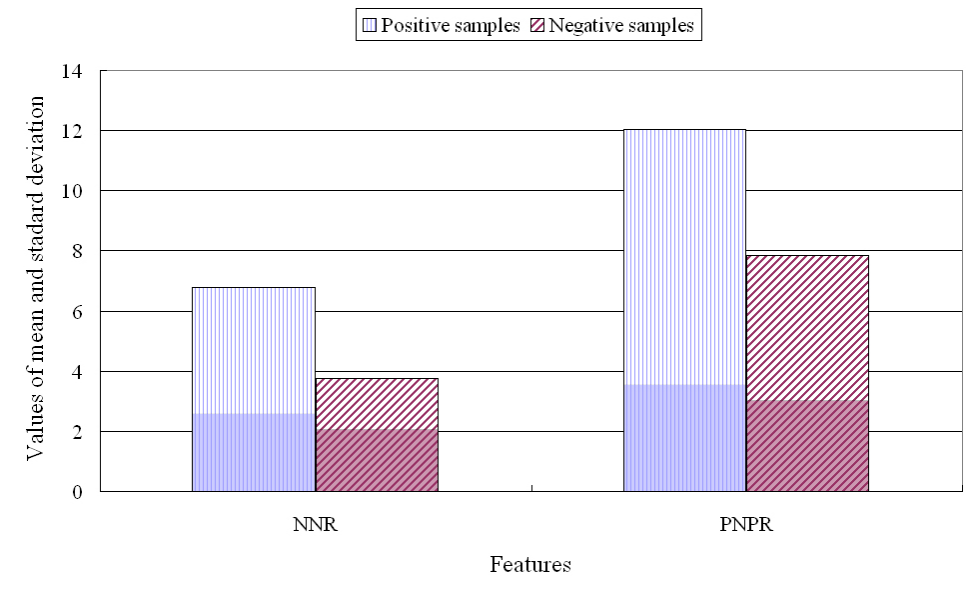
**Comparison of three features used for classification of transcription versus negative proteins**.

**Figure 4:**
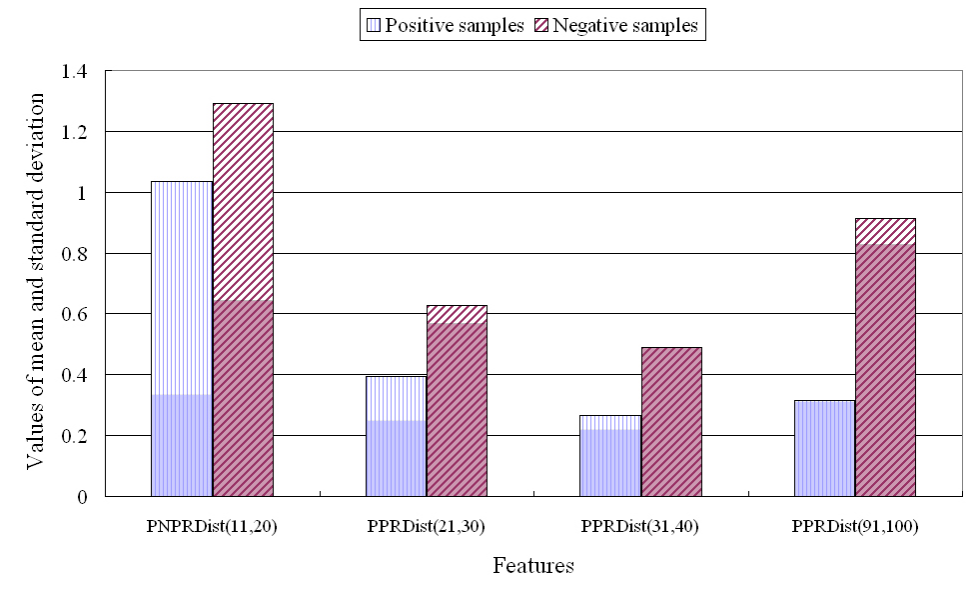
**Comparison of local information used for classification of gluconate utilisation versus negative proteins**.

The 451 traditional features used for prediction of protein function have been described in previous reports [17,22-24,28,32,36,37,43-45,76-78]. The present study introduces new features based on negatively and positively charged residues and analyses their utility. The average number of new features selected by CFS was 5.4 for the 11 protein classes. The raw dataset was analysed for the selected features, and three examples are provided in Figures 2, 3, and 4.

Figure 2 clearly demonstrates the differences in the means and standard deviations of the nine features used to classify guanine nucleotide-releasing factor (the opaque colour at the base of the bar graphs indicates the standard deviation). *PPR *and *NNR *for guanine nucleotide-releasing factor were higher than for the negative samples. For example, the *NNR *for guanine nucleotide-releasing factor was 7.04 (mean) ± 1.72 (standard deviation), while the *NNR *for the negative samples was 3.79 ± 2.0. It is worth noting that negatively charged residues appear more frequently in the guanine nucleotide-releasing factor sequence than in those of other proteins. Furthermore, the *NNR *and *PPR *features are related to the number or percentage of negatively and positively charged residues, as these features were computed using a method based on charged residues. Because of this relationship, the mean percentages of positively charged residues and negatively charged residues were found to be similar to the *PPR *and *NNR *values, respectively. If the *PPR *for a specific protein family was high compared to that for other families, then the number of positively charged residues in that protein family was also higher than that in other families; similarly, if the *NNR *for a specific protein family was low, then the number of negatively charged residues in that protein family was also low. However, if the percentage of negatively charged residues was high and the *NNR *value was low, it is possible to infer that both negatively and positively charged residues are present in the sequences, because *NNR *and *PPR *provide information on whether the two charged residue types co-exist in the sequence. The greater the difference between the percent negatively charged residues and the *NNR*, the more frequent is the alternating occurrences of negatively and positively charged residues. For instance, *golgi transport protein 1 *[Swiss-Prot:Q9USJ2] consists of 129 amino acids with five negatively charged residues and eight positively charged residues. However, *NNR *and *PPR *were 0 and 2.32, respectively. This indicates that although the protein includes five negatively charged residues, the positively and negatively charged residues in the sequence always alternate among the neutral residues.

Previous studies in this field have analysed only the number or percentage of positively and negatively charged residues; however, the positions or regions of the charged residues in the sequence are very important in determining protein function and structure [79-82]. For example, Verma *et al*. [79] analysed a large panel of plaque-purified recovered viruses and demonstrated that the negatively charged residues at positions 440 and 441 were key residues that appeared to be involved in virus assembly. Therefore, although the total number of positively and negatively charged residues is important, residues in specific positions or local regions of the sequences are also important. *Dist*_(*x*, *y*) _for *PPR*, *NNR*, and *PNPR *provides information on negatively and positively charged residues in local regions of the sequence. Seven features that provide local information were selected for classification of the guanine nucleotide-releasing factor. For example, *PNPRDist*_(61,70) _was 1.29 ± 0.42 for the positive classification samples and 0.72 ± 0.53 for the negative samples. These findings indicate that alternating positively and negatively charged residues occur more frequently in the local region from 61% to 70% in the guanine nucleotide-releasing factor sequence than in the negative protein samples. Therefore, local information on the distribution of negatively and positively charged residues in the interval was informative. Because of these essential differences, guanine nucleotide-releasing factor proteins can be predicted with a high level of accuracy.

Figure 3 presents the results of analysis of the raw data for two features used in classifying transcription proteins. *PNPR *for the positive classification samples was 12.04 ± 3.56, while for the negative samples, *PNPR *was 7.84 ± 3.03. These results indicate that continuous changes from a positively charged residue to the next negatively charged residue or vice versa occurred more frequently over the full sequence of transcription proteins than in the negative samples.

Figure 4 presents the results of analysis of the raw dataset for four features, when the proteins were classified using the random forest method. The mean values of the four selected features for gluconate utilisation were significantly lower than those of the negative samples. For example, *PNPRDist*_(11,20) _for the gluconate utilisation sequences was 1.03 ± 0.3, while *PNPRDist*_(11,20) _for the negative samples was 1.3 ± 0.6. These results indicate that there were fewer continuous changes from a positively charged residue to the next negatively charged residue or vice versa in the local region from 11% to 20% of the gluconate utilisation sequences than in the negative samples.

There are two ways of using positively charged residues in classification and prediction of protein function. One process uses the positively charged residues arginine (R), histidine (H), and lysine (K) [20], while the other uses only arginine (R) and lysine (K) [24]. Although defining two groups of positively charged residues is potentially useful, we found that the use of arginine, histidine, and lysine achieved better results than did arginine and lysine. Specifically, the former (R, H, and K) was useful in classifying gluconate utilisation, fatty acid metabolism, G-protein coupled receptor, transcription, and transport proteins, while the latter (R and K) was useful only in classifying fibre proteins.

### Significant findings

The following is a summary of the benchmark comparisons and important findings of this study for prediction of protein function over a broad range of cellular components, molecular functions, and biological processes. Analyses were conducted by SVM and the random forest method with and without feature selection, based on many traditional and proposed features extracted from the sequences.

• Using a larger number of features to predict protein function does not always result in improved performance. In terms of accuracy and AUC, SVM with feature selection has distinct performance advantages with this type of data, indicating that removal of the many redundant and irrelevant features by feature selection can improve prediction performance. However, there was no significant difference in prediction performance for the random forest method with and without feature selection.

• The use of a particular classifier does not always result in improved performance. There is no single method that is optimal for all conditions because the performance of a method depends on the type of data involved, the size of the dataset, the number of features involved, the type of extracted features, and whether feature selection is used, among other things. Therefore, selection of the optimal classifier for a given dataset depends on an understanding of machine-learning algorithms, feature selection processes, and biological background information relevant to the dataset.

• Features useful for predicting a specific protein function in a given dataset are not always useful for predicting another protein function – discriminative and informative features differ according to protein function. Therefore, identifying discriminative features applicable to a broad range of protein classes is difficult.

• Although many methods have recently been proposed for predicting protein function, most methods are not suitable for function prediction under high-throughput conditions, because they require information on protein structure. Currently, there is much more data available on protein sequences than on protein structures; thus, the methods developed in this study focused on predicting protein function based solely on features extracted from the protein sequence. This reduces the effort required to extract useful features, as the predictive or experimental work required to acquire structural information is both costly and time-consuming. In the experiments undertaken in this study, we found that sequence-based classifiers can also generate very good results.

• Local information regarding the protein sequence is meaningful in predicting protein function; several examples have been presented to demonstrate its usefulness. Although identifying local information for a sequence is difficult and the information does not always correspond to striking difference in protein function, unique features extracted from specific positions or local regions can be predicted with a high level of accuracy.

• The numbers or percentages of positively and negatively charged residues are some of the most important and well-known features used for function prediction. *PPR *and *NNR *were extracted from sequences based on the presence of negatively and positively charged residues. These novel features include information on the existence of negatively and positively charged residues as well as the manner in which the two charged residue types co-exist in a sequence. *PPR *and *NNR *were found to be selected more frequently for function prediction than were the number of negatively and positively charged residues. Thus, these features appear to be highly correlated with protein class and have a low inter-correlation with each other.

The above results indicate that it is possible to generate accurate predictions for a broad range of protein functions without the use of sequence or structural similarities. Feature selection improves predictions for a variety of protein functions, but does not always ensure improved performance, depending on the dataset and the method used. Finally, local information for protein sequences is meaningful for predicting protein function, and a feature set with good performance and dimensional reduction was identified, as many features initially included in this study were removed by CFS.

## Conclusion

Many previous studies have attempted to biologically and computationally determine meaningful and accurate features that assist in predicting protein function. Features that show an obvious propensity for predicting many different protein functions have not yet been reported, and this provides a motivation for discovering the relationship between features and protein function.

This paper described a highly accurate prediction method capable of identifying protein function by using features extracted solely from protein sequences, irrespective of sequence and structural similarities. In this study, the *PPR*, *NNR*, *PNPR*, and *Dist*_(*x*, *y*) _features were introduced. In predicting the functions of 11 different proteins, a high performance (94.23–100%) was achieved and predictive features for several protein classes were effectively identified.

The results presented here suggest that our new features, developed in the course of this study, will be useful in predicting many protein class functions. We believe that prediction performance can be improved by combining sequence-based features and additional features, such as predicted secondary structure, surface area, and subcellular location. Accordingly, further insight into feature analysis and biological understanding is needed. In future studies, we will apply this method to predict the functions of proteins that have not been identified by sequence alignment.

## Competing interests

The authors declare that they have no competing interests.

## Authors' contributions

BJL conducted the experiments and analysis, conceived the concepts of *PPR*, *NNR*, *PNPR*, and *Dist*_(*x*, *y*)_, and wrote the manuscript. MSS and YJO assisted in developing the method and revising the manuscript. KHR and HSO supervised the work, provided useful suggestions to improve performance, and revised the manuscript. All authors read and approved the manuscript.
